# Development of a balanced scorecard as a strategic performance measurement system for clinical radiology as a cost center

**DOI:** 10.1186/s13244-021-01009-2

**Published:** 2021-06-02

**Authors:** Ulf Teichgräber, Rainer Sibbel, Andreas Heinrich, Felix Güttler

**Affiliations:** 1 Institut für Diagnostische und Interventionelle Radiologie, University Hospital Jena, Friedrich-Schiller-University, Am Klinikum 1, 07747 Jena, Germany; 2 Frankfurt School of Finance, Frankfurt School of Finance and Management, Institute for International Health Management, Sonnemannstrasse 9-11, 60314 Frankfurt am Main, Germany

**Keywords:** Balanced scorecard, Key performance indicators, Controlling, Radiology, Management cockpit

## Abstract

**Objectives:**

To develop a goal-oriented indicator system based on the balanced scorecard (BSC) concept, which takes into account the perspectives of the referring physician and patient and emphasizes the focus on the internal processes of the radiology department.

**Methods:**

Development of a BSC occurred in six steps: (Step 1) strengths/weaknesses and opportunities/risks (SWOT-) analysis of the radiology department, (Step 2) setting-specific objectives (model, core values, key objective) followed by the development of 4 perspectives, (Step 3) and definition of strategic issues oriented to the value-added chain of the processes of the radiology department. (Step 4) Creation of a “Strategy Map” with regard to the perspective and their cause–effect relationships. (Step 5) Development of an automated key performance indicator (KPI) cockpit for the monitoring, reporting, and management scorecard.

**Results:**

A total of 10 success factors were identified using SWOT analysis. The core values include high quality in clinical, teaching, and research areas. The radiological value-added chain is composed of three processing steps. 1. registration, 2. examination, and 3. reading/X-ray demonstration. Three action programs were derived: 1. increase competency (e.g., specialist standard), 2. improve referring physician/patient satisfaction, 3. increase productivity. Daily process monitoring was added to the management cockpit as a monitoring scorecard. The scorecard comprises 18 KPIs and is automatically updated every month. The annual management scorecard comprises 10 KPIs.

**Conclusions:**

The BSC makes it possible to implement a strategy for radiology that is strongly oriented toward the requirements of the referring physicians and the demands of patients.

## Key points

- The implantation of a Balanced Scorecard concept in radiology represents a strategic management tool, which takes into account the perspectives of the referring physician and patient and emphasizes on the internal processes of the radiology department.
- Focusing on the value-added chain of the radiology department (process orientation) represents the fundamental idea of Balanced Scorecard for radiology.
- Balanced Scorecard based monitoring can help to overcome the weaknesses of a traditionally budget-oriented reporting system.

## Introduction

Radiology departments in a hospital function as a central internal cost center for other clinical departments and clinics (profit centers), such as the internal medicine, surgery, or emergency departments. As a rule, a radiology department usually is defined as a clinical institution with its own budget and cost responsibilities without its own wards (hospital beds) or outpatient clinics [1, 2]. Radiological services are reimbursed through internal service accounting. The current reporting system for radiology comprises a cost- and revenue-based system of indicators based on annual budgets. The planning and management of services provided are based solely on cost and revenue elements.

While the revenue side is closely related to the type and number of examinations performed, the cost structure in a radiology department is characterized by high personnel costs (as is typical for hospitals) on the one side and high technology-related, fixed costs on the other. Smooth organization of the processes with respect to personnel deployment, patient waiting times, and diagnosis ensures effective cooperation with requesting and referring clinics as well as the efficiency of the department. Therefore, the focus of management in the radiology department and the greatest optimization potential is in the design and organization of work processes. Traditional reporting in the radiology department does not consist of special measured values and key performance indicators (KPIs) that document and evaluate the work processes. Therefore, it is necessary to expand the current reporting system to focus on factors such as the work processes and use of resources and to be able to achieve goal-directed planning and management of added value for both patients and referrers as primary clients.

Kaplan/Norton developed a multidimensional system of indicators at the beginning of the 1990s known as the balanced scorecard (BSC) to serve as a management concept for implementing strategies. BSC is a concept for measuring, documenting, and managing the activities of a business or an organization with regard to the implementation of its vision and strategy with the intention of balancing the four perspectives of the services [3]. BSC is a link between strategy development and implementation. The traditional financial strategy targets in this concept are added to the operational objectives relating to the client, internal process, and resource-oriented learning and development perspectives. The entire value-added chain in this system of indicators is presented in line with cause/effect relationships in the four target fields (perspectives):

- Finances
- Market and clients
- Internal business processes/work processes
- Learning and growth (innovation)/potential

Therefore, the perspectives should not be considered independently but in a hierarchical structure according to Kaplan and Norton [4]. Among other things, the right balance of the resulting system of indicators is that the objectives and parameters that are related classically to financial results are related to the customer, process, and personnel factors and drivers that influence them significantly. Furthermore, quality-oriented “soft” success factors such as result quality, processing times, adherence to appointments, innovation, motivation, and qualification of employees can be evaluated along with “hard” fact-based KPIs.

In Health Care Organizations, it becomes more important that department heads serve as strategic managers with the ability to evaluate a changing industry, analyze data, question assumptions, and develop new ideas [5]. Kaplan and Norton also concerned themselves with the implementation of the BSC concept in nonprofit organizations (NPOs) and have published several case studies on this topic [6]. They determine that too little attention is paid to the intended output during strategy development. Hospitals in Germany also are operated predominantly as public law corporations or nonprofit corporations. For this reason, they are also regarded as NPOs. Kaplan and Norton summarize their experience with NPOs as follows: “BSC projects often were built on hastily implemented quality management systems that aimed to improve processes. It was difficult to find an NPO that followed a strategy which included product leadership and a close association with clients” [6].

**Fig. 1:**
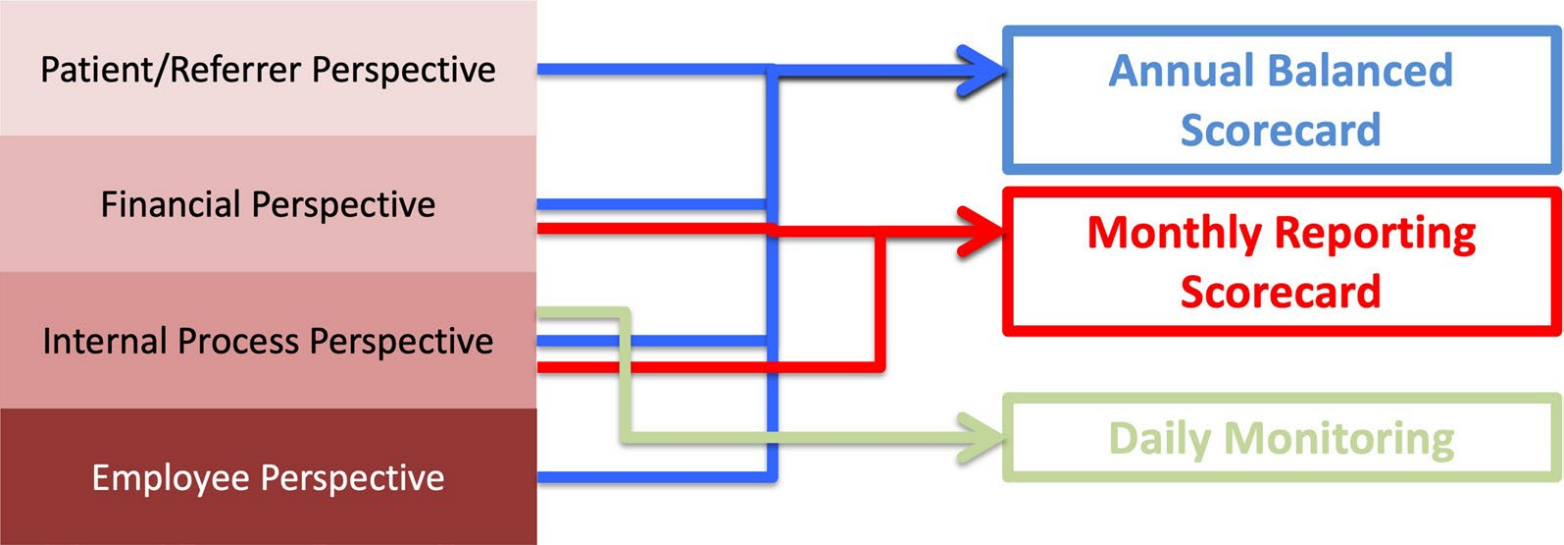
Deriving the scorecards from the four key perspectives

To address this lack of strategy-based management, Friedag and Schmidt have developed a highly practical approach to implementing BSC and have extended the concept to three consecutive scorecards (Fig. 1) [7]:

- A Daily Monitoring Scorecard depicts the current performance with the aid of process key values.
- A Monthly Reporting Scorecard replaces the monthly reporting system, summarizes the most important financial KPIs, adds to the internal processes (work processes) through use of KPIs, and links strategic management with operative controlling.
- An annual balanced scorecard serves as the basis for an annual reporting, summarizing all perspectives and used for strategic management.

For the radiology department as the internal cost center of the hospital, there is a large potential for improvement both in the sense of quality and the cost efficiency of internal processes. Therefore, internal monitoring of work processes (e.g., determining examination times, headways, patient waiting times, examination revenue) offers itself as an addition to the report and annual balanced scorecards. Daily monitoring is conducive to operative management of the department. The results of process monitoring are included in the report and annual balanced scorecards in an aggregate form.

## Methods

**Fig. 2:**
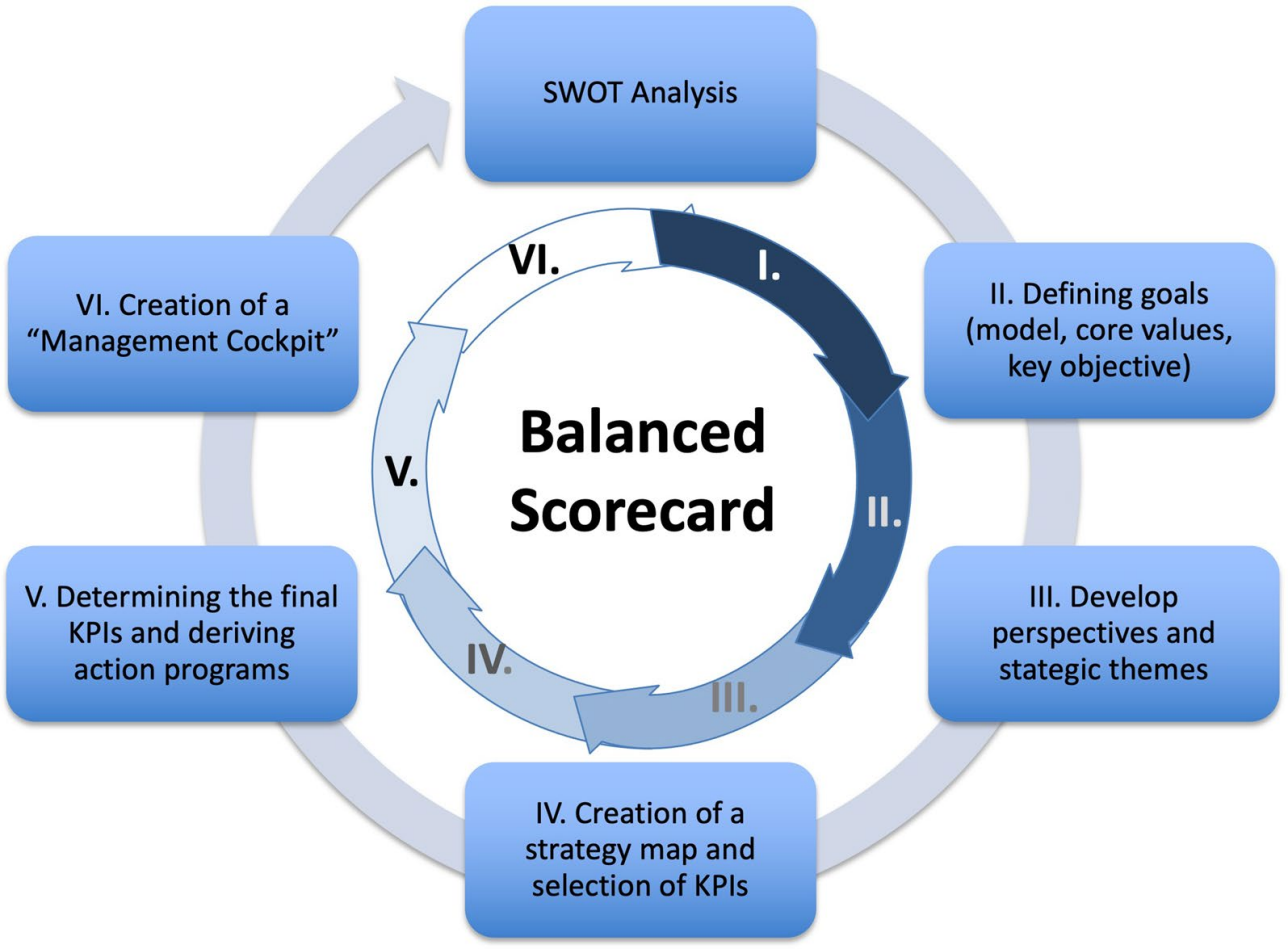
Process steps to create a balanced scorecard for the radiology department

The development of the BSC for the radiology department at our university hospital and its service portfolio occurred over six steps (Fig. 2): (Step 1) The first step included a strengths/weaknesses and opportunities/risks (SWOT-) analysis for internal and external current state analysis of the radiology department as a cost center within the university hospital with regard to internal and external influencing factors and changes. The SWOT matrix below presents the opportunities and risks analyzed in relation to identified strengths and weaknesses to define the limits for subsequent, long-term-oriented departmental strategy.

The second implementation step involved determining and defining the vision and the specific strategic objectives for the radiology department (model, fundamental values, key objective). The derivation of the key objective was performed through analyses of uniqueness, target clients, and the needs of their clients. The third implementation step was the development of four perspectives of the BSC design as well as the strategic areas of activity in the perspectives. The classical hierarchical arrangement of the perspectives according to Kaplan/Norton was modified in terms of the environment and for use in the radiology department. Instead of the financial perspective, the client perspective in terms of a cost center and the medical result dimensions was chosen as the highest hierarchical level. Unlike other clinical institutions in the hospital, the radiology department serves two categories of clients: patients and clinical referrers (other clinics within the hospital). We grouped both client categories as part of the client perspective, creating the patient and referrer perspective. This was then followed by the financial perspective, the internal process perspective and, finally, the employee perspective.

Strategic themes for each level based on this BSC structure were defined and oriented on the value-added chain of the processes in the radiology department. The radiological value-added chain consists of three basic process steps: 1. patient registration and the requested examination, 2. realization of the examination, and 3. findings/X-ray demonstration. Important time-dependent measured values in terms of process efficiency linked to these core processes were identified in a subsequent implementation step; these are closely linked to quality and profitability requirements on the client and financial levels.

**Fig. 3:**
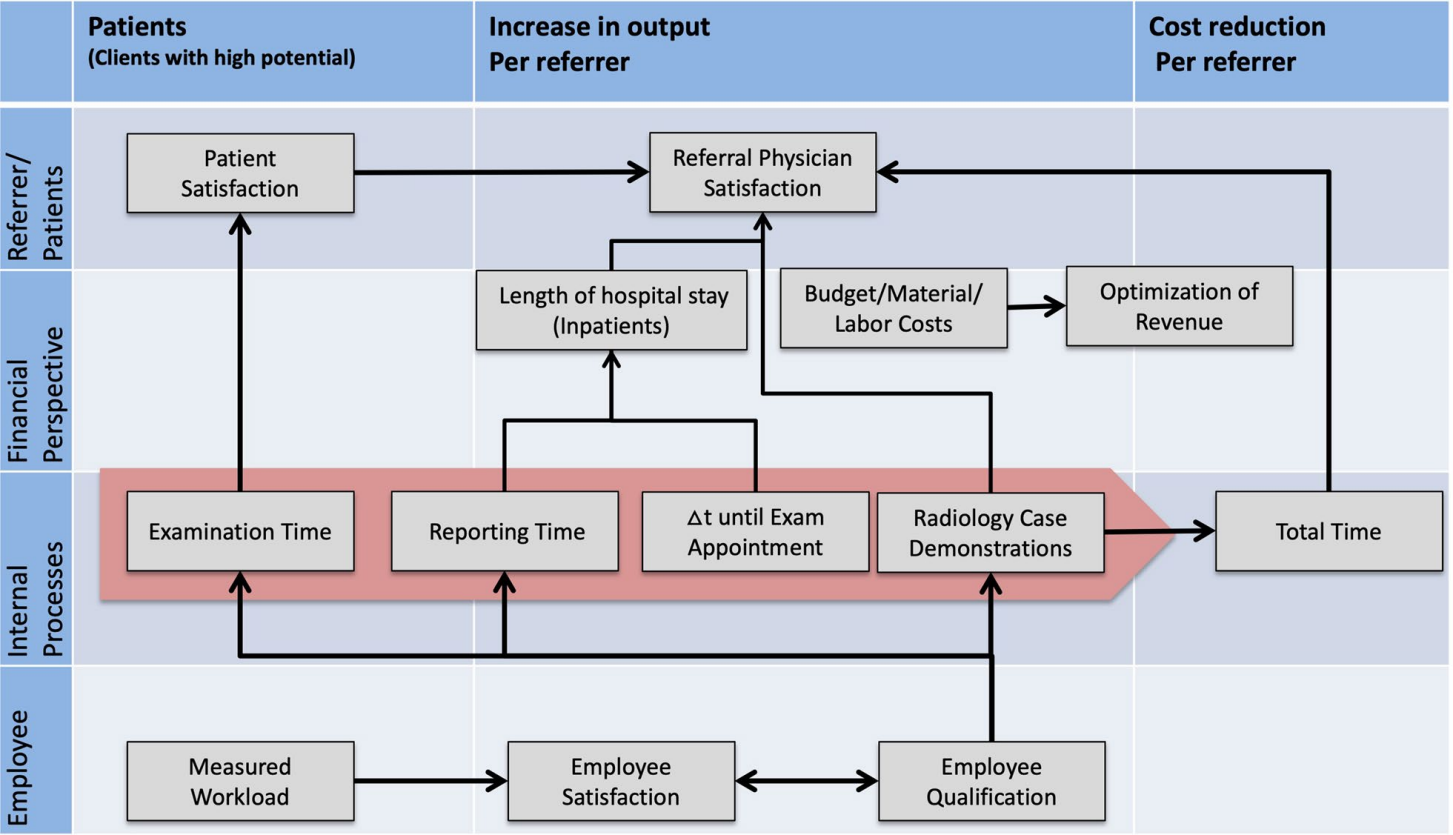
Strategy map of the radiology department

The focus of the fourth implementation steps was the creation of a strategy map (Fig. 3), which presents the cause/effect relationships below between the strategic areas of activity in the perspectives as well as subsequent definition and selection of KPIs based on the measured values for each area of activity.

Next, potential KPIs based on the thematic focus as well as the area of activity regarding the other perspectives and their cause/effect relationships were identified, which could be consulted with objective-oriented assessment of each core aspect. Possible process KPIs could be derived, e.g., from automatically measurement timestamps from the radiological information system (RIS). In total, nine different timestamps were collected. Additional KPIs were collected through literature research. For the referrer and employee perspective, KPIs from the radiology department annual electronic referral and employee survey were available.

For the subsequent selection of KPIs for the BSC, a unique evaluation concept with the acronym CSTC was developed. Based on four quality criteria, all previously compiled KPIs were evaluated and prioritized using a numerical ranking scale based on Concision, Survey effort, Topicality and Clarity. The fifth implementation step consisted of a final selection and definition of KPIs using a Horváth and Partner filter as well as the derivation of action programs [8]. In the sixth implementation step, the KPI cockpit for the monitoring, report, and annual balanced scorecard was created. The respective scorecards should be created completely automatically and be viewed at any time over a web portal. The annual balanced scorecard and the monthly reporting scorecard should be updated monthly and daily internal process monitoring pursued.

## Results

SWOT analysis resulted in a complete picture of the current situation as well as the important challenges for the hospital and the radiology department as a basis for strategic planning (Table 1). From this, a total of 10 success factors for a sustainably successful strategic positioning could be identified. Radiology as a cost center was defined as a departmental model. The fundamental values include high quality in clinical, teaching, and research areas. The vision, as well as the key objective in the long term, is to be in the top 10 in terms of innovation and services amongst German university radiology departments. The strategic issues discussed are part of the value-added chain of the radiological work processes and are also the most important performance drivers. From the strategy map along with a systematic literature analysis, 34 KPIs could be extracted for the radiology department and the CSTC evaluation conducted. The number of KPIs for the monthly reporting scorecard could be reduced to 18 using the Horváth and Partner filter. As a result, three action programs for the radiology department were created. 1. increase in competency (specialist standard, MTRA training), 2. improvement in referring physician/patient satisfaction, 3. increase in productivity daily process monitoring was added to the management cockpit as a monitoring scorecard. The monthly reporting scorecard consists of 18 KPIs and is automatically updated on a monthly basis. The annual management scorecard comprises 10 KPIs, each of which has a KPI from the annual electronic employee and referrer survey (Table 2).

**Table 1:** Strengths/weaknesses and opportunities/risks (SWOT-) analysis

Strengths	Weaknesses
*Internal analysis*	
Cost center for all radiological services within hospital	Lack of specialists, unfavorable ratio of young professionals to specialists during training
Wide range of additional training with complete training authority and additional designations for neuroradiology and pediatric radiology	
	High workload for the medical staff
	Long waiting times for examination appointments
	Long processing times
	Communication deficits with internal referrers
Modern organizational management with department system that has a matrix structure	
	No hospital beds in department for interventional radiological procedures
Highly specialized MRI and IR services	
Established research section for MRI physics, experimental radiology, and medical engineering	

Opportunities	Risks
*External analysis*	
Only university radiology department in the region	Competition through expansion of other hospitals into highly specialized services
Good public image through symposiums and congresses (regional, national, and international presence)	
	Good PR work through radiological CME events at other hospitals
Specialized diagnostic examinations and minimally invasive procedures are unique selling points	
	Lacking radiological medical care center for ambulatory services
	Most modern equipment in hospital for general and standard care
Modern instrumentation	

CME: continuing medical education, IR: interventional radiology, MRI: magnetic resonance imaging, PR: public relations

**Table 2:** Annual management scorecard for the radiology department

Annual balanced scorecard	
Perspectives	Key performance indicators
Referrer and patient perspective	*Appointment availability* Period until earliest possible appointment (Inpatients)
	*Patient waiting time* Waiting time on location (in the department)
	*Referrer satisfaction* Referrer survey
Financial perspective	*Technician productivity* Services/costs (technicians)
	*Medical staff productivity* No. of exams/costs (staff)
Process perspective	*Registration* △t from request to assignment of appointments
	*Release of reports* △t from examination to release of radiology report
	*Patient delay* Proportion of delayed patients
Employee perspective	*Additional training standard* Status of specialist training
	*Employee satisfaction* Employee survey

## Discussion

Mainline for-profit companies accepted BSCs in the meantime, but non-for-profit healthcare organizations were slow to adopt them for use. A number of problems face the healthcare industry, including cost structure, payor limitations and constraints, and performance and quality issues that require changes in how healthcare organizations, both profit and nonprofit, manage operations [9]. Already in 1998, Sahney demonstrated an approach how to apply a BSC in managed care organizations (MGOs) for measurement, development of strategy, and performance improvement. MGOs as healthcare providers are constantly facing the pressure of improving their financial performance in a highly competitive environment [10]. One of the first implementations of the BSC concept in the clinical environment was initiated by the Yale University School of Medicine in the field of Anaesthesiology published in 1999 demonstrating a great value to a department, even if the full implementation takes several years to complete [11]. In different healthcare sectors, the BSC concept is applied to support improved decision making and performance [12–17]. In order to attain a useful BSC in the healthcare sector, appropriate performance perspectives and indicators are crucial to reflect all strategies of the organization. It also requires medical staff to contribute in BSC development, which will result in greater reliability of the BSC [18].

In peer-reviewed journals in the medical field, there have only been two studies published on the application of BSC in a radiology department. An article from Mauer et al. presents a conceptual BSC for an academic university radiology department with focusing on teaching and research [19]. In this study, additional KPIs for teaching evaluation and examination grades were added to patient and referrer satisfaction from the client perspective. Additionally, five additional perspectives were suggested as a scientific perspective, which takes into consideration scientific publication output (number, impact factor) and the external funding secured. Academic university hospitals are often expected to excel in multiple domains: quality improvement, patient safety, education, research, administration, and clinical care [20]. The consideration of teaching and research in university medicine appears to be implied. In our present BSC, these important core tasks of the university radiology department have been ignored for the time being. Instead, the focus was placed on the clinical work processes to guarantee clarity and to limit where possible the number of KPIs in the BSC. In addition, at our university hospital, there is separate accounting for cost centers/budgets between clinical patient care and teaching and research. For that reason, it does not appear to make sense at present to take teaching and research into account in the same BSC. Rather, a second independent report and annual BSC should be established for teaching and research in parallel.

Donnelly et al. [21] present a case study for a BSC for a radiology department in a children’s hospital in the US Midwest. In this BSC, 33 KPIs in six perspectives were identified and designated as the department scorecard. The six perspectives are clinical service, teaching, research, patients, financial, and referrers. The BSC is created every quarter and available to all employees through an internet portal. It should be noted that the authors provided no cause–effect relationships between the KPIs. No information about the perspective hierarchy could be determined. The authors believe that the transparency of the KPIs for employees is essential to its use. They believe there is a motivation factor in the BSC for the employees, which is more effective than a financial incentive. The authors believe that establishing the BSC is an important instrument for quality improvement. The authors have noted improvement in 61% of KPIs, consistency in 33% of results, and a decline in only 6% of results over the 7 years since the implementation of the BSC [21].

The BSC concept allows for the development and implementation of a strategy for the radiology department, which is closely aligned with the requirements of the referring in- and outpatient clinics on the department as a cost center and considers patients to be the true clients. The BSC makes available a new management instrument for the radiology department of the Jena University Hospital with a measurable target not only to use existing potential efficiently but also to develop new potentials. The methodology of our BSC used and adjusted for the radiology department is not a cookbook recipe that can be used immediately for other clinical institutions and should always be adjusted methodically to align with the mission and the vision of each institution or hospital. Focusing on the value-added chain of the radiology department (process orientation) represents the fundamental idea of BSC for radiology. This approach can serve as a guide for other radiology departments at other hospitals when establishing a BSC. Individual KPIs can also certainly be used without modification. In these cases, it should be tested whether these align with the strategy and success factors of each institution. It is not recommended to simply adopt KPIs even within the same specialization.

After the implementation of our BSC, the different KPIs were used and accepted in different ways. The process KPIs showed their effect in daily monitoring. In addition, these process KPIs are made available to the internal referring clinical departments in monthly reports for the use of radiological services. KPIs such as the waiting time for examination appointments or radiological reports are the focus of interest. The annual BSC is used exclusively for the business planning and serves as an overview for the board of directors of our hospital for the operational results of the radiology department. For the personnel requirement calculation, the total performance figures, the utilization of the devices, and the minute-by-minute staff retention time of radiologists and technicians are used.

A strategic situation analysis including defining long-term-oriented objectives should always be the first step before defining KPIs for a BSC. The selection of KPIs should be conducted in reference to set-thematic focuses and a quality evaluation that also takes into account the survey effort and consequent practical feasibility for KPIs. An important stipulation when establishing a BSC for the Jena University Hospital radiology department was that a paper-based survey of KPIs would not be used. The KPIs survey should be completely automated. The results of the BSC would be communicated through a web portal in an electronic management cockpit. That ensured that the survey for the BSC in the radiology department would not and will not require additional personnel from the management division. At Jena University Hospital, the process and financial KPIs are all generated fully automatically via database queries. The Human Resource department data, on the other hand, must still be entered manually. In our experience, these KPIs are unfortunately not regularly updated, such as the level of illness. As a result, the personal KPIs can only be meaningfully evaluated within the framework of the annual BSC. This clearly shows how important automatic digital data transmission is for a BSC in order to use it effectively as a management tool.

## Conclusions

Overall, this example of the radiology department shows that BSC-based monitoring can also help hospitals to overcome the weaknesses of a traditionally budget-oriented reporting system and to be able to use different scorecards to meet the different requirements of a goal-oriented strategic and operational management.

## Data Availability

The data are included in the manuscript.

## Abbreviations

BSC: Balanced scorecard
CSTC: Concision, survey effort, topicality and clarity
KPI: Key performance indicator
MRI: Magnetic resonance imaging
SWOT: Strengths/weaknesses and opportunities/risks

## References

[1] Muchantef K, Forman HP (2005). Cost accounting in radiology: new directions and importance for policy. AJR Am J Roentgenol.

[2] Donnelly LF, Lee GM, Sharek PJ (2018). Costs of quality and safety in radiology. Radiographics.

[3] Kaplan RS, Norton DP (1992). The balanced scorecard–measures that drive performance. Harv Bus Rev.

[4] Kaplan RS, Norton DP (1996). Translating strategy into action—the balanced scorecard.

[5] Ginter PM, Duncan JW, Swayne LE (2018). Strategic management of health care organizations.

[6] Kaplan RS, Norton DP (2000). The strategy-focused organization: how balanced scorecard companies thrive in the new business environment.

[7] Friedag HR, Schmidt W (2000). My Balanced Scorecard: das Praxisbuch für Ihre individuelle Lösung.

[8] HaPM C (2020). Controlling concept—the: cornerstone of performance management.

[9] Pink GH, McKillop I, Schraa EG, Preyra C, Montgomery C, Baker GR (2001). Creating a balanced scorecard for a hospital system. J Health Care Finance.

[10] Sahney VK (1998). Balanced scorecard as a framework for driving performance in managed care organizations. Manag Care Q.

[11] Rimar S, Garstka SJ (1999). The "Balanced Scorecard": development and implementation in an academic clinical department. Acad Med.

[12] Fields SA, Cohen D (2011). Performance enhancement using a balanced scorecard in a Patient-centered Medical Home. Fam Med.

[13] Yeh TM, Lai HP (2015). Evaluating the effectiveness of implementing quality management practices in the medical industry. J Nutr Health Aging.

[14] Demartini C, Trucco S (2017). Are performance measurement systems useful? Perceptions from health care. BMC Health Serv Res.

[15] Wu X, Li S, Xu N, Wu D, Zhang X (2019). Establishing a balanced scorecard measurement system for integrated care organizations in China. Int J Health Plann Manag.

[16] Harvey HB, Sotardi ST (2018). Key performance indicators and the balanced scorecard. J Am Coll Radiol.

[17] Catuogno S, Arena C, Saggese S, Sarto F (2017). Balanced performance measurement in research hospitals: the participative case study of a haematology department. BMC Health Serv Res.

[18] Behrouzi F, Shaharoun AM, Ma'aram A (2014). Applications of the balanced scorecard for strategic management and performance measurement in the health sector. Aust Health Rev.

[19] Maurer MH, Teichgraber U, Kroncke TJ, Hamm B, Lemke AJ (2012). The balanced scorecard–applications in a radiology department. Rofo.

[20] Hwa M, Sharpe BA, Wachter RM (2013). Development and implementation of a balanced scorecard in an academic hospitalist group. J Hosp Med.

[21] Donnelly LF, Gessner KE, Dickerson JM (2010). Quality initiatives: department scorecard: a tool to help drive imaging care delivery performance. Radiographics.

